# Genetic and Transcriptomic Variations for Amoxicillin Resistance in *Helicobacter pylori* under Cryopreservation

**DOI:** 10.3390/pathogens10060676

**Published:** 2021-05-30

**Authors:** Xiurui Han, Yiyao Zhang, Lihua He, Ruyue Fan, Lu Sun, Dongjie Fan, Yanan Gong, Xiaoli Chen, Yuanhai You, Fei Zhao, Maojun Zhang, Jianzhong Zhang

**Affiliations:** State Key Laboratory of Infectious Disease Prevention and Control, Collaborative Innovation Center for Diagnosis and Treatment of Infectious Diseases, National Institute for Communicable Disease Control and Prevention, Chinese Center for Disease Control and Prevention, Beijing 102206, China; hanxiurui211@163.com (X.H.); zhangyiyaoicdc@foxmail.com (Y.Z.); helihua@icdc.cn (L.H.); fryforever@163.com (R.F.); sunlugogo@outlook.com (L.S.); fandongjie@icdc.cn (D.F.); gongyanan1024@126.com (Y.G.); 15165117996@163.com (X.C.); youyuanhai@icdc.cn (Y.Y.); zhaofei@icdc.cn (F.Z.); zhangmaojun@icdc.cn (M.Z.)

**Keywords:** *Helicobacter pylori*, *pbp1*, cryopreservation, amoxicillin resistance, transcriptome, plasma membrane

## Abstract

Some amoxicillin-resistant strains of *H. pylori* show a sharp decrease in amoxicillin resistance after freezing. In China, most clinical gastric mucosal specimens are frozen and transported for isolation and drug susceptibility testing for *H. pylori*, which may lead to an underestimation of the amoxicillin resistance. The objective of this study is to investigated reasons for the decreased amoxicillin resistance after cryopreservation. A high-level amoxicillin-resistant clone (NX24r) was obtained through amoxicillin pressure screening. After cryopreservation at −80 °C for 3 months, the minimum inhibitory concentration (MIC) of NX24r was reduced sharply. Mutations and changes of transcriptome were analyzed after amoxicillin screening and cryopreservation. Mutations in PBP1 (I370T, E428K, T556S) and HefC (M337K, L378F, D976V) were detected in NX24r, which may be the main reason for the induced amoxicillin resistance. No mutations were found in PBP1 or HefC after cryopreservation. However, transcriptome analysis showed that down-regulated genes in the cryopreserved clone were significantly enriched in plasma membrane (GO:0005886), including *lepB*, *secD*, *gluP*, *hp0871* and *hp1071*. These plasma membrane genes are involved in the biosynthesis and transport function of the membrane. The decreased amoxicillin resistance after cryopreservation may be related to the down-regulation of genes involved in membrane structure and transport function.

## 1. Introduction

*Helicobacter pylori* (*H. pylori*) infection is generally treated with a combination of proton pump inhibitors (PPI) and two or three antibiotics, with amoxicillin being one of the commonly used first-line antibiotics [1]. Amoxicillin resistance is associated with the treatment failure of *H. pylori* in regimens containing amoxicillin and the independent risk factor for treatment failure of clarithromycin-amoxicillin triple therapy [2]. Unsuccessful eradication therapies increase the resistance of *H. pylori* to antibiotics, including amoxicillin [3]. Guiding the rational use of antibiotics based on the results of drug susceptibility tests is conducive to increasing the eradication rate of *H. pylori* and reducing the bacterial resistance caused by the unreasonable use of antibiotics.

The eradication rate of *H. pylori* decreased in recent years with treatment of antibiotics including amoxicillin, but laboratory test results showed a low resistance rate of *H. pylori* to amoxicillin [4,5,6]. Notably, most gastric mucosa specimens are transported to laboratories under cryopreservation conditions before the isolation of *H. pylori* and drug susceptibility testing in China [4]. However, the resistance to amoxicillin of some *H. pylori* strains is unstable, and it is reduced substantially after cryopreservation, with some strains changing from a resistant phenotype to a susceptible phenotype [4,7]. These strains account for approximately 20–50% of amoxicillin-resistant *H. pylori* strains [8,9], which may lead to an underestimation of the amoxicillin resistance level of *H. pylori*. The production of beta-lactamase, changes in the structure of penicillin-binding protein 1 (PBP1), efflux pumps, and biofilm formation are all involved in the amoxicillin resistance mechanism of *H. pylori* [10,11,12,13], but whether these mechanisms are involved in the reduction of amoxicillin resistance level of *H. pylori* after cryopreservation is unclear.

This study examined the possible causes of reduced amoxicillin resistance in *H. pylori* by analyzing changes in the gene expression of unstable amoxicillin-resistant strains after cryopreservation.

## 2. Materials and Methods

### 2.1. Bacterial Strains and Culture Conditions

The *H. pylori* strain NX24 was isolated in our laboratory and identified by Gram staining microscopy, urease, catalase, and oxidase testing and 16S RNA sequencing. It was used as the initial strain to create the amoxicillin-resistant isolates. Karmali agar (Oxoid, Basingstoke, Hampshire, UK) supplemented with 5% defibrated sheep blood was used as basic medium for *H. pylori* culture. *H. pylori* isolates were incubated in a humidified microaerobic incubator (5% O_2,_ 10% CO_2_, 85% N_2_) at 37 °C.

### 2.2. Selection of Unstable Amoxicillin-Resistant H. pylori Strains In Vitro

The amoxicillin-resistant H. pylori isolates were obtained in vitro in our previous research [9]. Serial concentrations of amoxicillin were prepared in Karmali agar supplemented with 5% defibrated sheep blood. The initial concentration of amoxicillin for screening was 0.025 mg/L. Cultures were diluted to 10^9^ colony forming units per milliliter (CFU/mL) and inoculated with 100 μL and incubated at 37 °C in a humidified microaerobic incubator for 2–3 days. The screened *H. pylori* isolates were inoculated in a medium with a twofold concentration of amoxicillin to obtain stronger amoxicillin resistance. Isolates were purified and saved at various time points. The amoxicillin MICs for isolates were determined by Etest (Abbiodisk, Stockholm, Sweden). After 6 months of amoxicillin pressure screening, a high-level amoxicillin-resistant clone NX24r was obtained. It was preserved in brain heart infusion with 200 mL/L glycerol at −80 °C for three months then inoculated on Karmali agar medium supplemented with 5% defibrated sheep blood. The MICs to amoxicillin were determined after two passages to test whether the amoxicillin resistance is stable.

### 2.3. Polymerase Chain Reaction (PCR) and DNA Sequence Analysis

To determine the amino acid substitution of the obtained amoxicillin-resistant clone, the full length of genes encoding penicillin binding protein 1 (*pbp1*), TEM β-lactam (*tem*), porin protein (*hopB*, *hopC*) and efflux pump (*hefC*) were amplified by PCRand the DNA sequence was analyzed. Primers for PCR reaction are shown in Table 1. DNA was extracted from bacteria on a blood agar plate using a genomic DNA extraction kit (QIAGEN, Hilden, North Rhine-Westphalia Germany).

### 2.4. RNA Extraction and Library Construction

To explore the clues for the decrease of amoxicillin resistance of *H. pylori* after cryopreservation, transcriptome analysis was carried out on the original strain (NX24), the obtained amoxicillin-resistant clone (NX24r), and the cryopreserved clone (NX24f). Bacteria cultured for 48 h were suspended in 1 mL of Trizol reagent (Life Technologies, Carlsbad, CA, USA) and vibrated for 15 s. The suspension stood at room temperature for 5 min followed by centrifugation (12,000× *g*, 10 min, 4 °C). A 0.2 volume of chloroform was added to the supernatant, shaken for several seconds, left standing at room temperature for 3 min, and then centrifuged (12,000× *g*, 10 min, 4 °C). The supernatant was mixed with 0.5 mL isopropanol, left standing for 10 min at 4 °C, and centrifuged (12000× *g*, 10 min, 4 °C), and RNA was in the pellet. The pellet was washed twice with 1 mL of precooled 750 mL/L ethanol and centrifuged (12,000× *g*, 10 min, 4 °C). The solution was left at room temperature for 10 min to remove any residual ethanol. The pellet was dissolved in deionized diethyl pyrocarbonate (DEPC) treated water, incubated at 55 °C for 5 min, and stored at −80 °C for further use. Three biological replicates were performed for each clone, and the mean values represented the gene expression of the strain.

The quality of RNA was monitored using agarose gels, and the RNA concentration was measured using a Qubit RNA Assay Kit in a Qubit 2.0 Fluorometer (Life Technologies, Carlsbad, CA, USA). Sequencing libraries were generated using the NEBNext Ultra Directional RNA Library Prep Kit for Illumina (NEB, Ipswich, MA, USA).

### 2.5. RNA Sequencing and Data Pretreatment

The constructed libraries were sequenced using an Illumina Hiseq platform. Clean data were obtained by removing reads containing adapters, bases of low quality (Q score < 30) or short sequences (<20 bp). The filtered sequences were mapped and annotated in Bowtie2 software using *H. pylori* strain 26695 as the reference genome. Mapping to the reference genome and the annotation of transcripts were based on *H. pylori* strain 26695 using Bowtie2-2.2.3. HTSeq v0.6.1 was used to count the read numbers mapped to each gene, and the expression level of genes was calculated as the expected number of fragments per kilobase of transcript sequence per millions base pairs sequenced (FPKM).

### 2.6. Differential Expression Analysis and Functional Analysis

A differential expression analysis of genes between each amoxicillin-resistant strain (Three biological replicates) and the cryopreserved strains was performed using the DESeq R package (1.18.0), which provides a statistical model for determining differential gene expression based on the negative binomial distribution. Genes with a *p* value < 0.05 and |log_2_ (Fold change)| > 1 were assigned as differentially expressed. Gene ontology (GO) and kyoto encyclopedia of genes and genomes (KEGG) enrichment were performed to analyze the function of differentially expressed genes in DAVID 6.8 software (https://david.ncifcrf.gov/) accessed on 26 April 2021. GO and KEGG terms with a corrected *p* value (FDR) less than 0.05 were considered significantly enriched differentially expressed genes.

### 2.7. Quantitative Real-Time PCR

Total RNA served as a template for cDNA synthesis using the HiFiScript gDNA Removal RT MasterMix (Cwbio, Beijing, China) according to the manufacturer’s instructions. The qRT-PCR was performed with the primers shown in Table 2 by MagicSYBR Mixture (Cwbio, Beijing, China) to evaluate gene expression. HP1010, encoding the polyphosphate kinase (*ppk*), served as a housekeeping gene for qRT-PCR experiments [14]. Three replicates for each gene were evaluated, and the results in this paper represent the averages of at least three separate experiments.

## 3. Results

### 3.1. Mutation of PBP1, β-lactamase, Efflux Pump and Membrane Protein in Amoxicillin Resistant H. pylori NX24r

The high-level amoxicillin-resistant strain NX24r was obtained from the original sensitive isolate NX24 by in vitro pressure screening. The MIC value of NX24r was 256 mg/L.

We sequenced the β-lactamase gene *tem*, penicillin binding protein 1 *pbp1*, resistance-regulated cell division (RND) efflux pump gene *hefC*, and outer membrane porins *hopB* and *hopC* in NX24, NX24r and NX24f. The gene *tem* encoding β-lactamase was not detected in these isolates. Mutations of PBP1 (I370T, E428K, T556S) and HefC (M337K, L378F, D976V) were detected in NX24r and these mutations maintained a stable existence in NX24f after cryopreservation. No mutation in HopB or HopC was detected in the NX24r or NX24f (Table 3).

### 3.2. Changes of Transcription in NX24r after Amoxicillin Screening

**Figure 1:**
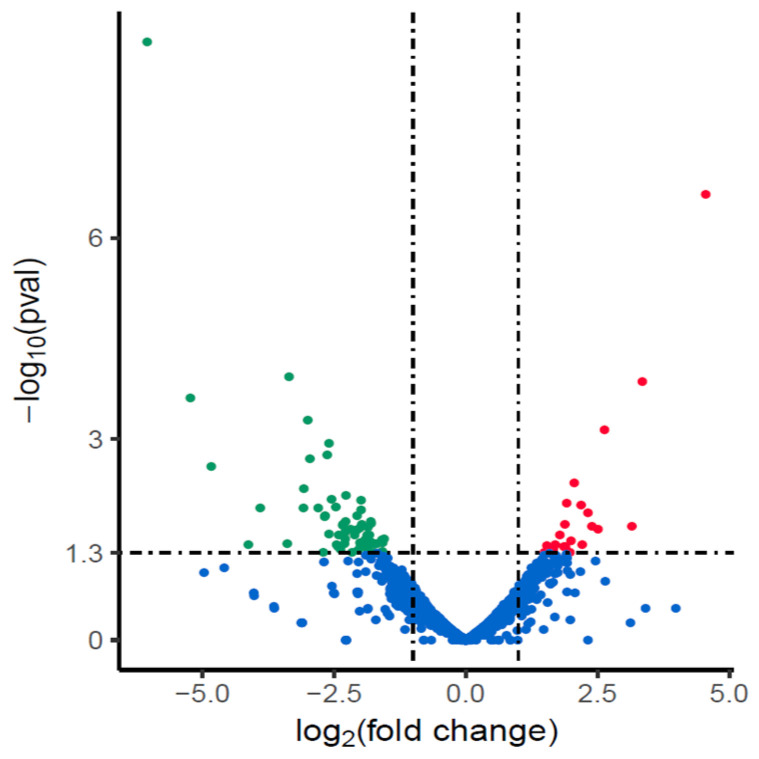
Differentially expressed genes in amoxicillin-resistant NX24r compared with NX24. Genes found to be up-regulated, down-regulated or unchanged in NX24r compared with those in NX24 in EdgeR analysis are represented as red, green and blue dots, respectively.

We compared the changes of gene transcription between amoxicillin-resistant NX24r and NX24. Data analysis revealed that 88 genes were differentially expressed (log2 fold change >1 or <−1) in the NX24r, and among them, 23 were up-regulated and 65 were down-regulated (Figure 1).

We analyzed the expression of *pbp1*, RND efflux family including *hefABC* (HP0605-HP0607), *hefDEF* (HP0971-HP0969), *hefGHI* (HP1327-HP1329), HP1489-HP1487 and outer membrane porin *hopB*, *hopC,* showed no change in the expression of all these genes.

GO clustering was used to analyze the function of differentially expressed genes in NX24r. However, the GO analysis did not provide significant terms.

### 3.3. Changes in Gene Expression of Cryopreserved H. pylori Isolate NX24f Compared with NX24r

The amoxicillin-resistant isolate NX24r was frozen at −80 °C. Three months later the MIC value of cryopreserved isolated (NX24f) was decreased to 5 mg/L, while those genes we sequenced did not mutate in NX24f compared with those in NX24r (Table 3).

**Figure 2:**
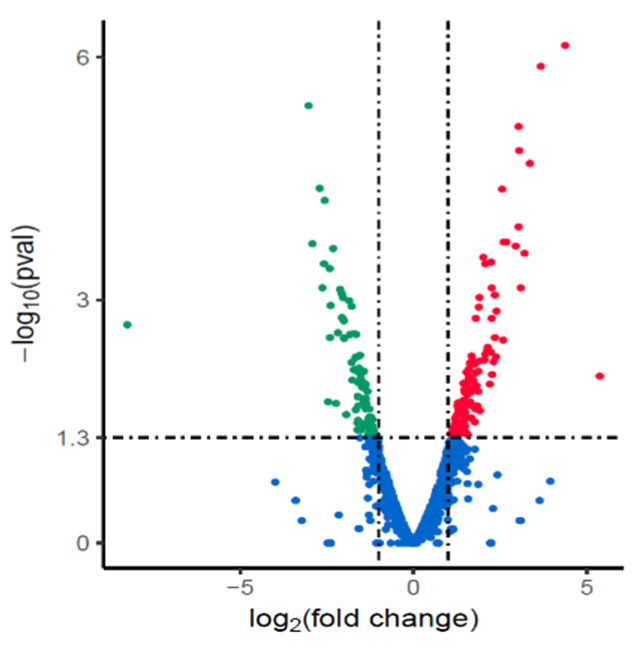
Differentially expressed genes in cryopreserved *H. pylori* isolate NX24f compared with NX24r. Genes found to be up-regulated, down-regulated or unchanged in NX24f compared with those in NX24r in EdgeR analysis are represented as red, green, and blue dots, respectively.

Transcriptomes between the NX24f and NX24r were analyzed to investigate the possible reason for the reduction in MIC caused by cryopreservation. The results showed that 199 genes were differentially expressed in the NX24f, including 107 up-regulated genes and 92 down-regulated genes (Figure 2).

There was no significant change in the expression of RND efflux pump genes in cryopreserved isolate NX24f.

GO clustering was used to analyze the function of genes differentially expressed in NX24f. The results showed that down-regulated genes were significantly enriched in the term of plasma membrane (GO:0005886) (FDR = 0.000818), while up-regulated genes were not enriched in any GO terms, suggesting that the membrane of *H. pylori* may change after cryopreservation. These 19 genes included in the plasma membrane are listed in Table 4. KEGG pathway analysis for these membrane genes did not find significantly enriched pathways.

### 3.4. Verifying the Expression Level of Plasma Membrane Genes by qRT-PCR

**Figure 3:**
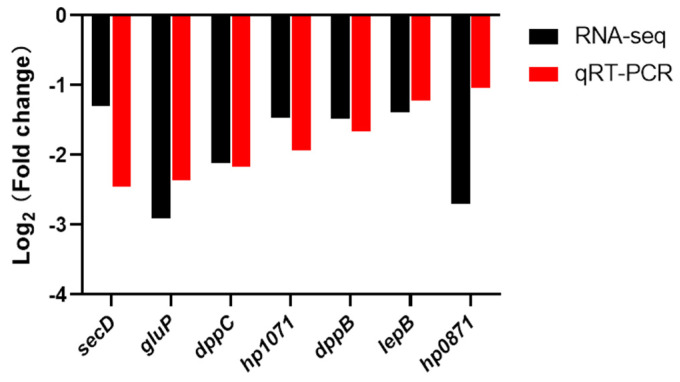
qRT-PCR analysis of the expression levels of plasma membrane genes in cryopreserved *H. pylori* isolate NX24f compared with NX24r.

We analyzed the difference in expression of some genes belonging to the plasma membrane between *H. pylori* NX24f and NX24r by qRT-PCR (Figure 3). As shown in Figure 3, The results of qRT-PCR showed that the expression of these genes was down-regulated in NX24f compared with that in NX24r, which was consistent with the results of RNA-seq analysis. The genes *hp0871* and *hp1071* are involved in the biosynthesis of glycerophospholipid, which is the main component of bacterial outer membrane. The genes *gluP*, *dppB*, *dppC*, *lepB* and *secD* are involved in the transport function of the membrane.

## 4. Discussion

In this study, a *H. pylori* isolate NX24r with high-level resistance to amoxicillin was obtained by amoxicillin screening in vitro. We analyzed the changes of gene mutation and transcription level during the screening process of amoxicillin and cryopreservation.

The resistance of Gram-negative bacteria to amoxicillin is usually caused by the following mechanisms: β-lactamase production, changes of PBP1, decrease of membrane permeability and bacterial efflux pump [15]. Although the production of β-lactamase is one of the main reasons for amoxicillin resistance, some studies have shown that there is no β-lactamase activity in *H. pylori* with high amoxicillin resistance [16,17,18]. No β-lactamase encoding gene was detected in this study, indicating that the amoxicillin resistance of NX24f was not due to β-lactamases.

Amoxicillin inhibits the synthesis of cell wall by binding with PBPs, thus preventing the growth of bacteria. It has been reported that the mutations of the *pbp1* gene confer amoxicillin resistance to *H. pylori* [19,20]. The PBP1 protein has acyl transpeptidase activity, and this domain contains three putative penicillin binding motifs: SXXK_338–368_, SXN_236–559_ and KTG_555_. S402G in the second PBP motif, E406A, S417T, S414R, T555S and N561Y substitutions in the third PBP motif are the main reasons of amoxicillin resistance in *H. pylori* strains. Univariate analysis of mutation sites also shows that S414R in PBP1 is related to amoxicillin resistance [11,20]. In this study, mutations of PBP1 were detected in NX24r (I370K, E428K, T556S), which are located at or adjacent to the third PBP motif, suggesting mutations in the PBP1 may be related to the amoxicillin resistance of NX24r.

Effluent pump and membrane permeability are also important reasons for drug resistance of bacteria. In Gram-negative bacteria, the RND family is the representative of multi-drug resistant efflux pump. *H. pylori* contains three putative RND efflux systems: hefABC (HP0605-HP0607), hefDEF (HP0971-HP0969) and hefGHI (HP1327-HP1329) [21]. HefC is a component of the efflux pump hefABC, which depends on proton dynamics. Studies have shown that the mutation of HefC affects the resistance of *H. pylori* to metronidazole and amoxicillin [22]. The change of porin affects the permeability of the cell membrane, thus affecting the accumulation of antibiotics in bacteria. It was found that the accumulation of penicillin G in HopB and HopC mutant strains decreased by about 20% and 10%, respectively, compared with their parent strains [23]. In this study, we detected hefC mutations (M337K, L378F, D976V) in the amoxicillin-resistant clone NX24r, but no mutations were detected in porin HopB and HopC, and there was no significant change in the transcription of these genes. The transcriptome analysis showed that there was little difference in transcriptome between NX24r and NX24. This suggested that mutations of PBP1 and efflux pump may be the main reason for resistance of NX24r to amoxicillin. Kwon, D.H., et al. [18] transformed the strains with high resistance to β-lactam into susceptible strains by gene transformation, and found that the high resistance to β-lactam was the result of the change of PBP1 and the decrease of membrane permeability, which was consistent with our research.

Cryopreservation is a serious challenge to the survival of bacteria. *H. pylori* is extremely susceptible to cryopreservation compared to other gastrointestinal bacteria such as *Escherichia coli.* It cannot survive when stored in saline at 4 °C, −20 °C or −80 °C for 3 weeks [24]. The number of surviving *H. pylori* after cryopreservation decreased by 50–90%, even with the protection of glycerin and sucrose [24,25]. The cryopreservation process not only caused the death of *H. pylori*, but also significantly reduced the drug resistance of some amoxicillin-resistant *H. pylori*. Han, S.R. [8] reported that the MIC decreased to <0.016 μg/mL after cryopreservation in three of seven amoxicillin-resistant *H. pylori* strains (MIC > 256 μg/mL). In this study, the MIC of the high-level drug-resistant isolate decreased from 256 (NX24r) to 5 μg/mL (NX24f) after cryopreservation. There were no mutations found in the cryopreserved NX24f in *pbp1*, *hefC*, *hopB* and *hopC*, suggesting that the decreased amoxicillin resistance after cryopreservation may be not caused by gene mutation.

The bacterial membrane separates bacteria from the external environment and is involved in the adaptation of bacteria to environmental stresses such as low temperature and antibiotics [26,27,28]. Studies have shown that the membrane is the main target of damage in the process of bacterial freezing [29]. Genes related to membrane protein regulation are down-regulated during the repair of sublethal *Staphylococcus aureus* after cryopreservation [30]. Unsaturated fatty acids can increase the fluidity of cell membrane, and their content affects the tolerance of bacteria to low temperature. However, the content of unsaturated fatty acids in cell membrane of *H. pylori* is very low [31,32], which may be the reason *H. pylori* is particularly susceptible to cryopreservation. In the freezing process, ice is first formed in the bacteria preservation solution, and most solutes are transferred to the unfrozen part of the preservation solution, causing an increased external osmotic pressure of bacteria and dehydration of bacteria and contraction of plasma membrane. The plasma membrane can cope with this contraction by endocytosis to form vesicles and reduce plasma membrane area. However, the reduction of membrane structure beyond a certain degree is irreversible and is difficult to recover, which may cause cell rupture during osmotic expansion in thawing. GO analysis showed that the down-regulated genes of *H. pylori* were significantly enriched in plasma membrane (GO:0005886) after cryopreservation in NX24f. These down-regulated membrane components were related to membrane proteins, lipoproteins and transporters (Table 4), which may reflect the changes in the structure and function of the membrane after cryopreservation.

The outer membrane of Gram-negative bacteria acts as a selective barricade, the inner leaflet of which is composed of glycerophospholipids and the outer leaflet is composed of lipopolysaccharide (LPS) [33]. Its structural change is an important reason for antibiotic resistance [34]. It has been shown that changing the permeability of the outer membrane can reverse bacterial resistance to amoxicillin in amoxicillin-resistant Escherichia coli [35]. The gene *hp0871* and *hp1071* encode the CDP-diacylglycerol pyrophosphatase and CDP-diacylglycerol-serine O-phosphatidyltransferase, respectively, which are involved in biosynthesis of glycerophospholipid. The deletion of CDP-diacylglycerol pyrophosphatase causes the accumulation of UDP-containing LPS intermediates and inhibits the synthesis of LPS [36]. Proteins destined for the outer membrane are synthesized with a signal sequence that is cleaved by the signal peptidase I LepB for integral outer membrane proteins when they cross the cytoplasmic membrane [37]. LepB is considered to be a new target for the treatment of Gram-negative bacteria. Inhibition of LepB activity reduces the resistance of *Pseudomonas aeruginosa* to colistin and other antibiotics [38]. SecD is a member of the Sec transport family and participates in the synthesis and transport of lipoproteins. In Escherichia coli, the mutation of the SecD gene affects the modification of the protein with glyceride and causes the export defect of outer membrane lipoprotein [39,40]. We also detected other plasma membrane genes related to transport function including *dppB*, *dppC* and *gluP*. The dipeptide ABC transporter is responsible for transporting dipeptides and some oligopeptides, which is important for the growth of *H. pylori* [41]. The genes *dppB* and *dppC* encode dipeptide ABC transporter permease, while dppD and dppF encode ATP binding domain. The destruction of dipeptide ABC transporter significantly reduces the peptide use ability of *H. pylori* and causes deficiencies in bacterial growth [42,43]. In addition, Miftahussurur, M. [44] found that there were multiple site mutations in *dppB* in metronidazole-resistant *H. pylori* strains. The gene *hp1174* encodes the glucose/galactose transporter (GluP), which is an efflux pump of the major facilitator superfamily (MFS) [45]. GluP is involved in the uptake of D-glucose and the synthesis of *H. pylori* biofilm matrix, which may limit the transport of antibiotics to bacteria [46,47]. Ge, X. [14] found that the expression of *gluP* in multi-drug resistant *H. pylori* strains was significantly higher than that in sensitive strains. The down-regulation of these genes may lead to changes in the structure and permeability of the outer membrane of bacteria, thus affecting the tolerance of *H. pylori* to amoxicillin.

In conclusion, we analyzed the gene mutations and transcriptome changes of amoxicillin-resistant *H. pylori* screened in vitro and analyzed the possible reasons for the decrease of amoxicillin resistance after cryopreservation. The mutation of PBP1 and efflux pump proteins including HefC may be the main reason of amoxicillin-resistant *H. pylori* induced in vitro. However, the decrease of amoxicillin resistance after cryopreservation may mainly be related to the down-regulation of genes involved in membrane structure and transport function. The results of amoxicillin susceptibility tests for cryopreserved *H. pylori* strains may not accurately reflect the true susceptibility of clinical strains. Clinical gastric mucosa specimens should be avoided cryopreservation before *H. pylori* isolation and drug susceptibility test. There are some limitations in our study. Only one *H. pylori* strain was used in this research, and the high-level amoxicillin-resistant *H. pylori* isolate in the research did not become susceptible after cryopreservation despite the sharp decrease in the MIC value. Therefore, our analysis is not a comparison between the amoxicillin-resistant isolate and amoxicillin-susceptible isolate.

## Figures and Tables

**Table 1:** Primer sets used for PCR in this study.

Gene	Primer	Sequence (5′-3′)
*pbp1*	pbp1-F	TGCATAAAGGCATTAGACAATCAAG
	pbp1-R	GCTATCCGCCCTCCTACGGT
*TEM*	TEM-F	ATAAAATTCTTGAAGACGAAA
	TEM-R	GACAGTTACCAATGCTTAATCA
*hefC*	hefC-F	ATGTATAAAACAGCGATTAATCGTCCTATTACGAC
	hefC-R	TCATTCTAAAGTTTTTTGGTTTTGATAAAACCGCTT
*hopC*	hopC-F	ATGATAAAGAAAAATAGAACGCTGTTTCTTAGT
	hopC-R	TTAGAATGAATACCCATAAGACCAATAAACG
*hopB*	hopB-F	ATGAAACAAAATTTAAAGCCATTCAAAATGAT
	hopB-R	TTAGAAGGCGTAGCCATAGACC

**Table 2:** Primer sets used for qRT-PCR in this study.

Gene	Primer	Sequence (5′–3′)
*hp1010*	HP1010_F	GCGCGTTAGTCGTTTATGGCGTTT
	HP1010_R	AGCGCTCAAAGGGTTGTAATTGCC
*hp0300*	HP0300_F	CGCTCCTTGGATGCTTGTT
	HP0300_R	CATGATGCCATCGCCTACC
*hp0576*	HP0576_F	GGGAGGGATAAACACCACCA
	HP0576_R	CGGCATACCCATTCCTAAAA
*hp0871*	HP0871_F	GTTTGTACGCATTAGGCACTTCTT
	HP0871_R	GTTTGAATGGGCATAAATACTTGG
*hp1071*	HP1071_F	GTGAGCAATATCCGCTACCCTA
	HP1071_R	TGCCATAAATCAAATACAACCC
*hp1550*	HP1550_F	ATCTGTAAGCATTTCGCCATCT
	HP1550_R	AAATTAGGCAGCGTGTTGTTGT
*hp1174*	HP1174_F	CCGCTGGTAATCCCTTTGTA
	HP1174_R	CTTGCATTATCGCCCATTTT
*hp0299*	HP0299_F	AGGAGATCCGGCGTTAGTGA
	HP0299_R	GCGTCAATAGCGGCTTGATT

**Table 3:** Mutations in PBP1, HefC, HopB and HopC in *H. pylori* isolates.

Isolates	MIC of Amoxicillin (mg/L)	PBP1	HefC	HopB	HopC
NX24 initial	<0.016				
NX24r	256	I370T, E428K, T556S	M337K, L378F, D976V	/	/
NX24f	5	I370T, E428K, T556S	M337K, L378F, D976V	/	/

**Table 4:** List of genes enriched in the plasma membrane.

Gene ID	log_2_FC	Gene Name	Description
HP1174	−2.915	*gluP*	glucose/galactose transporter
HP0871	−2.703	*hp0871*	CDP-diacylglycerol pyrophosphatase
HP1168	−2.625	*cstA*	carbon starvation protein
HP0300	−2.121	*dppC*	dipeptide ABC transporter permease
HP0226	−2.034	*hp0226*	membrane protein
HP0942	−1.823	*hp0942*	D-alanine/glycine permease
HP0724	−1.575	*hp0724*	anaerobic C4-dicarboxylate transporter
HP1069	−1.557	*ftsH*	cell division protein
HP0770	−1.506	*flhB*	flagellar biosynthesis protein
HP0299	−1.485	*dppB*	dipeptide ABC transporter permease
HP1071	−1.461	*hp1071*	CDP-diacylglycerol--serine O-phosphatidyltransferase
HP0576	−1.384	*lepB*	signal peptidase I
HP1571	−1.369	*rlpA*	rare lipoprotein A
HP0943	−1.326	*dadA*	D-amino acid dehydrogenase
HP0888	−1.296	*hp0888*	iron chelatin transport ATP-binding protein
HP1270	−1.295	*hp1270*	NADH-quinone oxidoreductase subunit K
HP1550	−1.295	*secD*	preprotein translocase subunit
HP1041	−1.233	*flhA*	flagellar biosynthesis protein
HP0791	−1.137	*hp0791*	cadmium, zinc and cobalt-transporting ATPase

## Data Availability

All RNA-Seq raw data have been deposited in the China National Microbiological Data Center (accession number NMDC10017701 and transcriptome accession numbers NMDC40001387 to NMDC40001389) and can be found at: http://nmdc.cn/resource/genomics/sra?keyword=NMDC10017701 (accessed on 2 December 2020).
